# Role of Deregulated microRNAs in Breast Cancer Progression Using FFPE Tissue

**DOI:** 10.1371/journal.pone.0054213

**Published:** 2013-01-23

**Authors:** Liang Chen, Youhuai Li, Yebo Fu, Jin Peng, Meng-Hsuan Mo, Michael Stamatakos, Christine B. Teal, Rachel F. Brem, Alexander Stojadinovic, Michael Grinkemeyer, Timothy A. McCaffrey, Yan-gao Man, Sidney W. Fu

**Affiliations:** 1 Division of Genomic Medicine, Department of Medicine, Department of Microbiology, Immunology and Tropical Medicine, The George Washington University School of Medicine and Health Sciences, Washington, District of Colombia, United States of America; 2 Breast Cancer Division, Department of Surgery, Baoji Central Hospital, Baoji, Shaanxi, China; 3 Department of Pathology, The George Washington University School of Medicine and Health Sciences, Washington, District of Colombia, United States of America; 4 Department of Surgery, The George Washington University School of Medicine and Health Sciences, Washington, District of Colombia, United States of America; 5 Department of Radiology, The George Washington University School of Medicine and Health Sciences, Washington, District of Colombia, United States of America; 6 Surgical Oncology, Walter-Reed Army Medical Center, Washington, District of Colombia, United States of America; 7 The Diagnostic and Translational Research Center, Henry Jackson Foundation, Gaithersburg, Maryland, United States of America; George Mason University, United States of America

## Abstract

MicroRNAs (miRNAs) contribute to cancer initiation and progression by silencing the expression of their target genes, causing either mRNA molecule degradation or translational inhibition. Intraductal epithelial proliferations of the breast are histologically and clinically classified into normal, atypical ductal hyperplasia (ADH), ductal carcinoma *in situ* (DCIS) and invasive ductal carcinoma (IDC). To better understand the progression of ductal breast cancer development, we attempt to identify deregulated miRNAs in this process using Formalin-Fixed, Paraffin-Embedded (FFPE) tissues from breast cancer patients. Following tissue microdissection, we obtained 8 normal, 4 ADH, 6 DCIS and 7 IDC samples, which were subject to RNA isolation and miRNA expression profiling analysis. We found that miR-21, miR-200b/c, miR-141, and miR-183 were consistently up-regulated in ADH, DCIS and IDC compared to normal, while miR-557 was uniquely down-regulated in DCIS. Interestingly, the most significant miRNA deregulations occurred during the transition from normal to ADH. However, the data did not reveal a step-wise miRNA alteration among discrete steps along tumor progression, which is in accordance with previous reports of mRNA profiling of different stages of breast cancer. Furthermore, the expression of MSH2 and SMAD7, two important molecules involving TGF-β pathway, was restored following miR-21 knockdown in both MCF-7 and Hs578T breast cancer cells. In this study, we have not only identified a number of potential candidate miRNAs for breast cancer, but also found that deregulation of miRNA expression during breast tumorigenesis might be an early event since it occurred significantly during normal to ADH transition. Consequently, we have demonstrated the feasibility of miRNA expression profiling analysis using archived FFPE tissues, typically with rich clinical information, as a means of miRNA biomarker discovery.

## Introduction

Among women, breast cancer is the second most notorious cause of cancer deaths after lung cancer, and the most prevalent form of cancer, excluding skin cancer. According to the American Cancer Society, approximately 230,480 new cases of invasive breast cancer are expected to be diagnosed, as well as an estimated 57,650 additional cases of *in situ* tumor in the United States in 2011, and about 39,520 women are expected to die from the malignance. Current prognosis and treatment results vary depending upon the stage and size of the tumor; Ten-year disease-free survival rates vary from 98% to 10%. Early detection of the preneoplastic lesion remains the key to improving patient outcomes and survival, reducing patient suffering and costs. Therefore, more accurate and powerful diagnostic and predictive tools are needed for early non-invasive breast cancer.

Current concepts regard intraductal epithelial proliferations of breast as a heterogeneous disease composed of various types of neoplasms and subpopulations (e.g. hyperplasia, atypical hyperplasia, DCIS) in different areas of the same tumor. Thus, they differ in their potential to progress and metastasize. It is reasonable to postulate that there will be significant gene expression differences among different sub-populations in the same tumor tissue, all of which undergo hypoxia and hormone stimulation within the same microenvironment. However, most breast cancers would receive adjuvant chemotherapy and/or other interventions after diagnosis. Therefore it is hard to determine whether or not non-metastatic tumors were impacted by aggressive treatment, thus challenging the representativeness of the sampling.

Archival collections of Formalin-Fixed, Paraffin-Embedded (FFPE) tissues, linked to clinical databases, provide a rich and efficient resource for biological insight compared to collections of fresh frozen tissues. Because biomarkers developed from FFPE samples could be more rapidly translated into clinical practice, FFPE tissues can be an invaluable tool for biomarker discovery and validation [1]. The formalin fixation process allows for permanent preservation of the architecture of the tissue in optimal histological condition and easy long-term storage. Unfortunately, this process also compromises the yield, quality, and integrity of the nucleic acids through enzymatic and chemical degradation, extensive cross-linking with proteins and various chemical modifications [2] [3]. miRNAs, partially due to their size, are potentially more robust to FFPE-dependent degradation than mRNAs. Therefore miRNAs could be a viable alternative for expression profiling. Furthermore, it has been demonstrated in multiple studies that miRNAs are minimally affected by FFPE treatment, as isolated miRNAs displayed reliable expression levels as compared to frozen tissue samples [4] [5] [6] [7].

Although extensive research on molecular mechanisms involved in breast cancer has been done recently, challenges still prevail in the early diagnosis and management of breast cancer patients, such as unpredictable response and development of resistance to adjuvant therapies. miRNAs, as regulators of protein-coding genes, could serve as novel diagnostic and prognostic candidates, and thus as potential therapeutic targets. Recent studies have indicated that circulating miRNAs may serve as minimally invasive biomarkers for cancer diagnosis [8]. Since the deregulation of miRNA in breast cancer was first reported in 2005 [9], there have been many studies on the expression of various miRNAs and their roles in breast cancer (Table 1). miRNA profiling studies have led to the identification of miRNAs that are aberrantly expressed in human breast cancer, with miR-10b, miR-125b and miR-145 being down-regulated and miR-21 and 155 being up-regulated. Tumor formation may arise from miRNA deregulation. Iorio et al. [9] identified 29 miRNAs that were differentially expressed in breast cancer tissue compared to normal tissue, and a further set of 15 miRNAs that could be used to discriminate between tumor from normal cells. In addition, miRNA expression has been correlated with biopathological features such as ER and PR expression (*miR-30*) as well as tumor stages (*miR-213* and *miR-203*). Differential expression of several *let-7* isoforms was associated with PR status (*let-7c*), lymph node metastasis (*let-7f-1*, *let-7a-3*, *let-7a- 2*), or high proliferation index (*let-7c*, *let-7d*) in tumor samples. Mattie et al. identified unique sets of miRNAs associated with breast cancers currently defined by their HER2 or ER/PR status [10]. miRNAs exert their function by directly targeting downstream genes and their associated pathways with sequence preference on mRNA seed sequence [11]. They can function as either tumor suppressors [12]–[15] or oncogenes (sometimes refer to as oncomir) [16]–[23]. Thus, tumor formation, progression and metastasis may arise from a suppression of tumor suppressor miRNAs and/or overexpression of an oncogenic miRNA. We attempt to profile miRNA expression patterns to identify potential biomarkers for the diagnosis of pre-invasive breast lesions.

**Table 1:** Microdissected samples from breast cancer FFPE blocks.

Patients	Lesions			
	NORMAL	ADH	DCIS	IDC
A	A1*	X	A3	A4
B	B1	B2	B3	B4
C	C1	X	C3	C4
D	D1	D2	D3	X
E	E1	E2	X	E4
F	F1	X	F3	F4
G	G1	G2	X	G4
H	H1	X	X	H4

* The letters (A, B, C …) represent each patient and the numbers, 1, 2, 3, 4 indicate “Normal”, “ADH”, “DCIS”, “IDC” respectively in each patient's FFPE tissue. “X” means that no histological samples were obtained from an individual FFPE sample.

In this study, we identified 8-patient FFPE blocks that contain multiple components of the tissue, such as histologically normal epithelial, ADH, DCIS and/or invasive tumor cells. We microdissected each sample as described earlier [24] and collected tissue samples. Total RNAs were isolated for miRNA microarray analysis. We observed different miRNA expression patterns between different subgroups, which may allow us to identify unique miRNA signatures for each neoplasm type. After expression profiling, we obtained a list of miRNAs based on representatives from different clusters for discrete stages of classification: statistically significant expression levels were identified as from the 50^th^ percentile and upward; comparison to prior publications demonstrating their functional implications in breast cancer or other tumors; application of commercially available qRT-PCR assays for validation. To validate our findings, we performed a second microarray expression profiling assay on 16 patients with definitive diagnosis of normal, ADH, DCIS and IDC cases. Using the same criteria as described above, we obtained a unique list of miRNAs that are differentially expressed. Then, we extracted overlapping miRNAs from both studies. The expression of these miRNAs was further verified by TaqMan qRT-PCR. We identified molecular targets of these miRNAs using the target prediction analysis by three different algorithms, such as TargetScan 6.0, Diana microT 3.0 and miRanda (microRNA.org). As a proof of principle, we used anti-miR-21 oligo to transfect MCF-7 and Hs578T cells, and as predicted, we observed restoration of MSH2 and SMAD7 expression levels following miR-21 knock-down. MSH2 is a component of the post-replicative DNA mismatch repair system (MMR), frequently mutated in hereditary nonpolyposis colon cancer (HNPCC). SMAD7 is an antagonist of signaling by the TGF-β1 superfamily members and has been shown to inhibit TGF-β and activin signaling by associating with their receptors thus preventing SMAD2 access.

## Results

### Laser Capture Microdissection (LCM) Approach and FFPE total RNA Isolation

Breast cancer is a heterogeneous disease. To isolate the different components of the premalignant breast tissue during the breast cancer progression, we applied laser capture microdissection on 8 patient FFPE samples. Components of ADH, DCIS and IDC were collected when available in addition to the adjacent normal epithelium cells from all 8 patients. As expected, not all FFPE samples contain all lesion components (Table 1).

The ABI RecoverAll™ Total Nucleic Acid Isolation Kit for FFPE Tissues kits was used to isolate total RNA from the microdissected FFPE tissue following the protocol described in the Materials and Methods section. We routinely obtained more than 50 µg of total RNA from 4∼5 15 mm thick sections, with an OD 260/280 ratio≈2.0 and RIN (RNA Integrity Number) between 2.1∼2.4. The low RIN was expected due to the nature of FFPE fixation. However, it seems it has minimal adverse impact on miRNA analysis.

### MicroRNA Expression Comparisons in Early Breast Cancer

Using Agilent miRNA microarray technology, we profiled 24 LCM samples from 8 FFPE blocks, including 8 Normal, 4 ADH, 5 DCIS, and 7 IDC samples (Table 1). Sample D3 (DCIS sample from Patient D), was excluded for robust statistical analysis due to its failure to pass quality control. For unpaired analysis, miRNA microarray expression profiling was also performed on an additional 16 samples with clear clinical diagnoses without subjecting them to LCM.

We first performed paired comparisons with patient-matched histological types: ADH vs. Normal, DCIS vs. Normal, IDC vs. Normal and IDC vs. DCIS, by using the *t-*test module in the GeneSpring GX. Lists of differentially expressed miRNA candidates with statistical significance (*p*< = 0.05) are shown in Table 2. Differentially expressed miRNAs with *p*<0.05 and fold change of 2.0 or above were verified by real-time PCR. Four miRNAs (miR-21, miR-183, miR-200c and miR-200b) were significantly up-regulated when comparing ADH vs. normal. miR-21 has been well documented as an oncogene, while miR-200c/b are reported as biomarkers for primary hepatocellular carcinoma [25] and miR-200c as an independent prognostic factor in pancreatic cancer [26]. Most interestingly, when comparing DCIS vs. normal, we found 53 significantly changed miRNAs, including miR-195, which is a potential biomarker for noninvasive and early stage breast cancer in blood testing [27], and shows differential expression between DCIS and normal cells [28]. Comparing IDC and normal, miR-933, miR-141 and miR-96 were found to be altered significantly.

**Table 2:** A representative list of deregulated miRNA entities during the breast lesion transition.

Comparisons	miRNA IDs	*p* value	Regulation
**ADH vs. Normal**	hsa-miR-1275	0.011393113	DOWN
	**hsa-miR-638**	0.021915715	DOWN
	**hsa-miR-572**	0.02500332	DOWN
	**hsa-miR-671-5p**	0.025993915	DOWN
	**hsa-miR-21**	0.03355437	UP
	**hsa-miR-200b**	0.039687086	UP
	hsa-miR-15b	0.04428858	UP
	**hsa-miR-183**	0.044314582	UP
	hsa-miR-30d	0.049228158	UP
**DCIS vs. Normal**	hsa-miR-557	0.001621039	DOWN
	hsa-miR-1207-5p	0.008294453	DOWN
	hsa-miR-874	0.0190089	DOWN
	hsa-miR-556-3p	0.045900322	UP
**IDC vs. Normal**	**hsa-miR-638**	0.001237625	DOWN
	hsa-miR-575	0.002705719	DOWN
	hsa-let-7f	0.005910912	UP
	**hsa-miR-671-5p**	0.008136721	DOWN
	hsa-miR-20a	0.012225322	UP
	hsa-miR-15a	0.012381793	UP
	hsa-miR-1202	0.014062578	DOWN
	**hsa-miR-183**	0.016907487	UP
	hsa-miR-141	0.017054873	UP
	hsa-miR-19b	0.021237634	UP
	hsa-miR-1915	0.024006981	DOWN
	hsa-miR-107	0.02413377	UP
	**hsa-miR-21**	0.024726247	UP
	hsa-miR-1274b	0.025880286	UP
	hsa-miR-1268	0.027592959	DOWN
	**hsa-miR-200b**	0.028273659	UP
	hsa-miR-106b	0.03564651	UP
	hsa-miR-634	0.037061296	DOWN
	hsa-miR-129*	0.037617348	DOWN
	**hsa-miR-572**	0.04061733	DOWN
	hsa-miR-933	0.04227081	DOWN
	hsa-miR-17	0.042894967	UP
	hsa-miR-29b	0.0460609	UP
	hsa-miR-877*	0.04818575	UP
	hsa-miR-425	0.048827756	UP

A set of samples diagnosed with Normal, ADH, DCIS, and IDC (4 of each) were subject to the microarray analysis as we performed for the microdissected groups. In both paired and un-paired analyses, there were more deregulated miRNAs during the Normal-ADH transition compared to other processes. Deregulated miRNAs that appeared in both analyses are bolded.

Our unpaired microarray analysis from the second set of samples identified 74 miRNAs that were differentially expressed when comparing ADH to Normal, whereas DCIS and IDC showed no significance in miRNAs alteration (Supplementary 2). This is agreeable to our paired t-test analysis, which also shows more altered miRNAs in normal to ADH transition. Additionally, most of the miRNA deregulations (6 out of 9) that occurred in the transition of Normal-ADH were also observed in later stages. Taken together, the comparison results of the paired test indicate that miRNA alterations are more significant during the Normal-ADH transition than DCIS-IDC transition, and these alterations can be maintained into later stages, a report which has been confirmed by other investigations [29]. The most significant miRNA expression changes occurred at the early tumor initiation stage, suggesting that these miRNAs may serve as biomarkers for early breast cancer detection and management.

### Unsupervised Clustering on Different Clinicopathologic Samples with all Detected miRNAs

**Figure 1:**
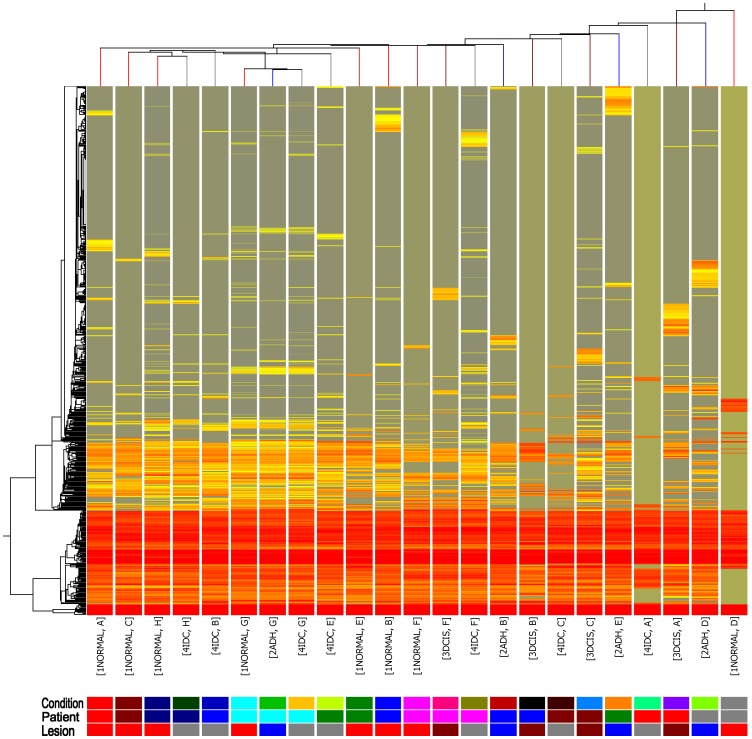
Unsupervised clustering results on both miRNAs and conditions of the 23 samples. One solid color box represents a certain condition. The clustering dendrogram indicates stages from the same patient were more closely clustered than those from the same stages.

It is widely believed that breast cancer initiates from the premalignant ADH stage and then cumulates into the potentially lethal IDC stage in a linear model. Therefore it is rational for us to hypothesize that there are unique stepwise broad-wide miRNA alterations for each stage transition. In other words, discrete pathological stages of early stage breast cancer could have broad-wide miRNA expression signatures. To pursue this hypothesis, unsupervised hierarchical clustering was carried on 23 distinct tumor stage samples and also on all detected miRNAs on the arrays using Euclid correlation and centroid linkage. However, after hierarchical clustering, we failed to readily find distinct clusters separated by different stages as expected. Instead, asynchronous stages from the same patient were shown to cluster more closely to each other than to their peer-stages from different patients (Fig. 1). This seems to be consistent with mRNA expression profiling in the progression of human breast cancer as previously reported [29]. This finding is also reasonable as the distinct stages of breast cancer are evolutionally associated with the same origin tumor colony or tumor stem cell within the individual patient. Therefore the alterations of most miRNA repertories are inherited from that stem cell and differ from others. Furthermore, it might also explain the reason why some patients diagnosed with ADH or DCIS never progress to IDC.

### MiRNAs as Potential Molecular Markers for Early Stage Breast Cancer

**Figure 2:**
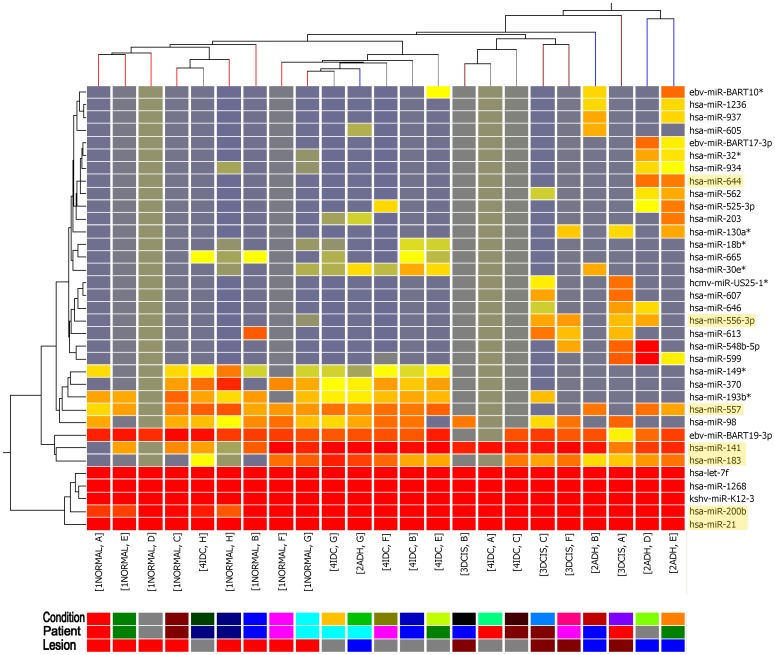
Unsupervised clustering results on ANOVA identified miRNAs and conditions of the 23 samples. Each solid color box represents a certain condition. The clustering result indicates the significantly altered miRNA entities identified by ANOVA test have more potential to distinguish different stages of breast cancer than broad-wide miRNAs. Seven individual clusters were clearly discerned by the clustering algorithms and the miRNAs circled by red rectangles representing their discrete clusters.

Instead of persisting on the existence of a stage specific miRNA signature of early breast lesion, we started to focus on whether there will be some individual or combination of unique miRNAs for each stage. ANOVA test was applied to look for stage specific deregulated miRNAs with statistical significance (*p*< = 0.05). We successfully found 35 miRNAs with unique expression in one certain stage against the others. Another unsupervised hierarchical clustering based on the identified differentially expressed miRNAs was generated to determine if they can distinguish between the different stages of breast lesion. The clustering results indicated the significantly altered miRNA entities identified by the ANOVA test distinguished between different stages of breast lesion better than broad-wide miRNAs. Seven individual clusters were clearly discerned by the clustering algorithms (Fig. 2). We selected a short list of miRNAs (miR-644, miR-556-3p, miR-557, miR-141, miR-183, miR-200b and miR-21) based on both their representation of different clusters for classification on discrete stages, as well as their higher expression levels and relevance to breast cancer.

### QRT-PCR Verification on Microarray Result

**Figure 3:**
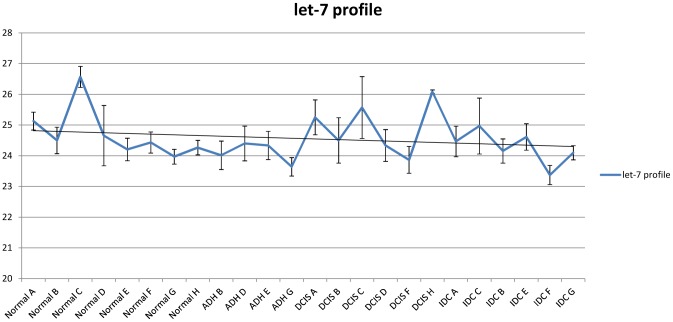
qRT-PCR results displaying the raw Ct values of let-7a across all samples and stages. The profile shows that let-7a expression was relatively consistent among different components and patients. The error bars indicate that the standard error of mean (SEM) is n = 4.

**Figure 4:**
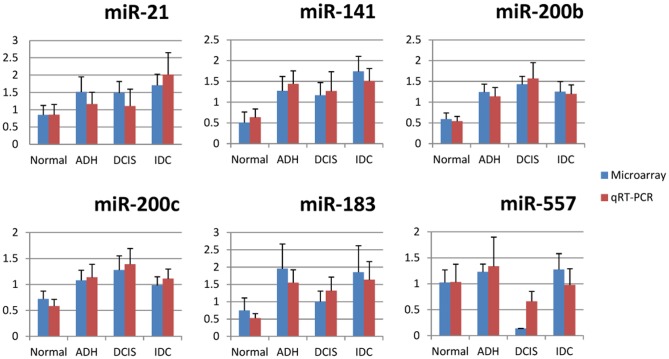
QRT-PCR verification of miRNAs expression results from microarray data. Blue bars represent the results from microarray, while red bars indicate the results from qRT-PCR. The error bars are the standard error of mean (SEM) for each analysis. QRT-PCR results are largely consistent with our microarray data. Five representative miRNAs (miR-21, miR-183, miR-141, and miR-200b/c) were observed up-regulated during the Normal-ADH transition, and their high expression levels were maintained throughout the tumor developmental stages. miR-557 was found to be down-regulated specifically in the DCIS stage.

QRT-PCR was performed to verify the miRNAs selected from the ANOVA test. We first chose miR-16 and let-7a as reference genes; however, let-7a was used as the sole reference subsequently since there appeared to be little difference between using both and using let-7a alone (Supplementary 3). Let-7a showed a comparatively consistent and abundant expression level within all the samples (Fig. 3). One of the identified up-regulated miRNAs, miR-200b, has been widely reported as a tumor suppressor miRNAs. To verify this, we also assessed the expression of miR-200c, which belongs to the same miR-200 family. It is believed that miRNAs from the same family might have similar expression patterns. Furthermore, our second unpaired microarray also showed increased expression levels of miR-200b (data not shown). We observed that the expression of miR-141, miR-183, miR-200b/c and miR-21 began to increase during the Normal-ADH transition, and maintained their high expression profiles during later stages. Interestingly, low expression of miR-557 was observed in the DCIS stage (Fig. 4). The correlative expression results from qRT-PCR analysis were consistent with the expression pattern by microarray assays. However, miR-644 and miR-556-3p were hardly detectable by qRT-PCR, as raw Ct values were underneath the detectable baseline (data not shown). In summary, there seems to be promising evidence of a group of miRNAs that might have the potential to distinguish between discrete stages of breast cancer procession.

### Unpaired Analysis of miRNA Microarray Data

**Figure 5:**
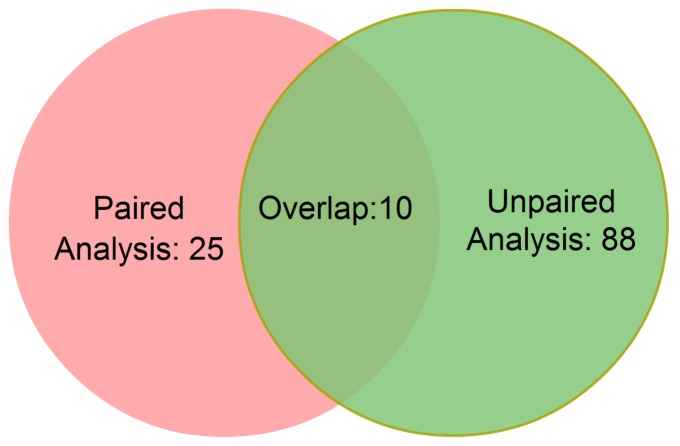
Venn diagram of ANOVA test results from paired and unpaired miRNA expression analysis. ANOVA test on the paired miRNA microarray data analysis resulted in 35 deregulated miRNAs, while ANOVA test on the unpaired analysis showed 98 deregulated miRNAs. There are 10 overlapping miRNAs (miR-1268, mir-130a, miR-141, miR-193b, miR-200b, miR-21, miR-320a, miR-370, miR-557 and kshv-mir-K12-3).

To confirm our initial findings, we performed miRNA microarray expression profiling on the 16 patient samples, including 4 normal, 4 ADH, 4 DCIS and 4 IDC. An ANOVA Benjamin and Hochberg FDR corrected test was performed to identify stage specific deregulated miRNAs. The expression of 98 miRNAs were identified as significantly altered (*p*< = 0.01) from this unpaired analysis, of which among them 10 were overlapped with our previous paired ANOVA analysis (Fig. 5). Therefore the 10 overlapped miRNAs may have significant value in both diagnosis and management of early breast cancer.

### Prediction of miRNAs' Target Genes and Pathway Analysis

We performed miRNA target prediction as well as their associated pathway analysis, using the three most common algorithms: TargetScan Human 6.0 (http://www.targetscan.org), Diana microT 3.0 (http://diana.cslab.ece.ntua.gr/microT/) and miRanda (http://www.microrna.org/microrna/home.do). Targets were regarded as positive only if they were predicted by at least two algorithms. The ones that were predicted by all three algorithms were bolded. Target gene lists were subjected to pathway analysis using GeneSpring GX and IPA (Ingenuity Pathway Analysis) for potential significant pathway analysis (Supplementary data 4). Of the identified pathways, the most significant pathway regulated by miR-21 was the TGF-β pathway. Target prediction results indicate that miR-21 might promote the TGF-β pathway by silencing the inhibitor SMAD7. An activated TGF-β pathway, therefore, can accelerate the generation of mature miR-21 in a feed-forward loop fashion. The TGF-β pathway was reported to have both a tumor promoting and suppressing effect. MiR-21 can debilitate its tumor suppressing branch by silencing MSH2, one of the DNA mismatch repair genes [30].

### MiR-21 Inhibition in Human Breast Cancer

**Figure 6:**
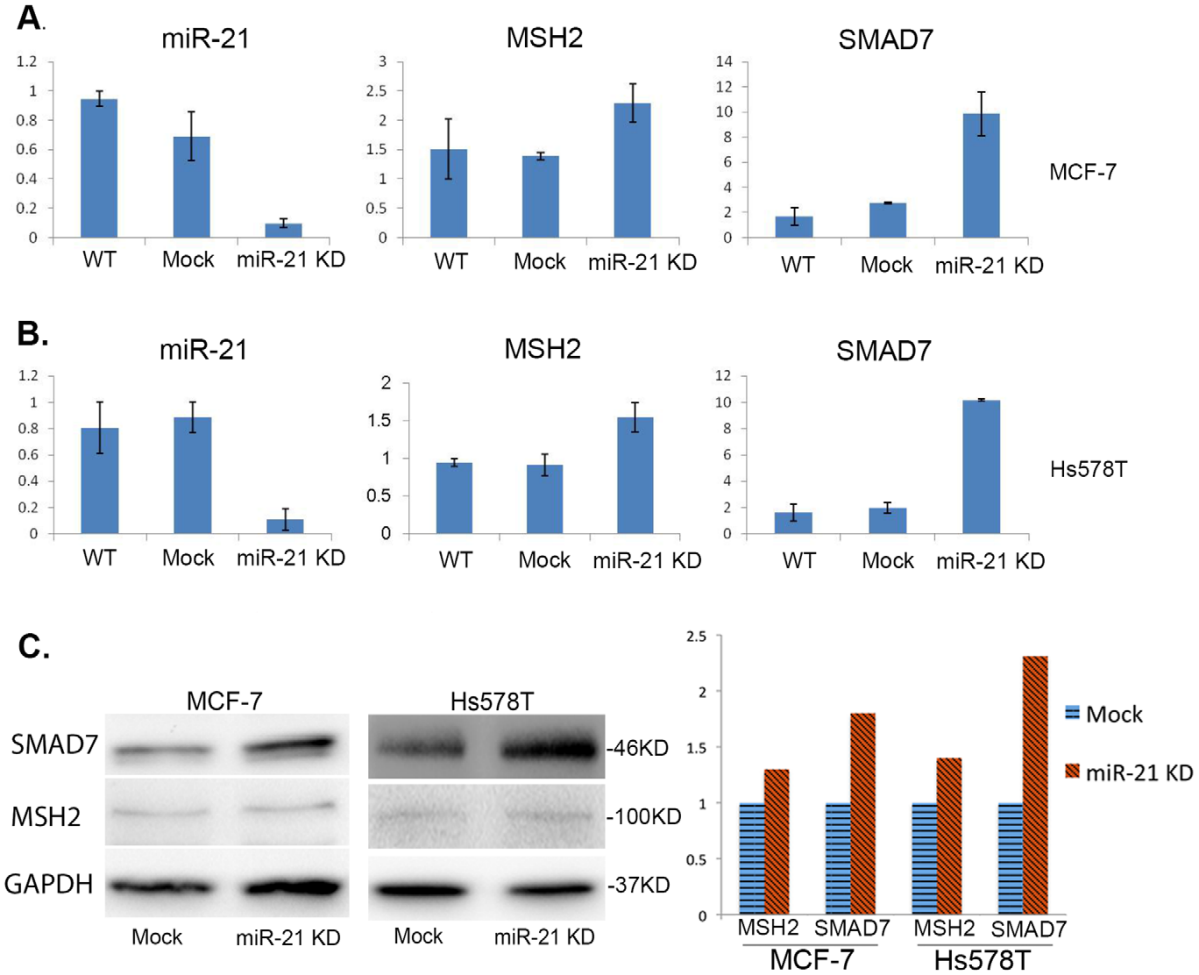
Knockdown of miR-21 restores the expression of SMAD7 and MSH2 in MCF-7 and Hs578T breast cancer cell lines. MCF-7 and Hs578T cells were transfected with miR-21 inhibitor and a negative mock control using the Lipofectamine 2000 kit (Invitrogen). After 48 hrs, miR-21 expression level was knocked down by ∼10 fold as compared to the mock controls in both MCF-7 (Fig. 6A) and Hs578T (Fig. 6B) cell lines using the Invitrogen SYBR green qRT-PCR kit. Untransfected cells were also included in the analysis (WT). With down-regulated miR-21 in both MCF-7 and Hs578T cells, MSH2 and SMAD7 mRNA expression was up-regulated by ∼1.67 and ∼3.6 fold, respectively (Fig. 6A and 6B), while the protein level was increased by ∼35–43% for MSH2 and ∼80–133% for SMAD7 (Fig. 6C).

MiR-21, one of the most reported miRNAs, is involved in the progression of many cancers. Our study shows during the early stage of breast lesion, miR-21might function as a major force in driving tumor progression, due to its continuously high expression level. TGF-β signaling is well studied for its anti-mitogenic function during the early stages of cancer, but promotes invasion and metastasis in later stages. Activated TGF-β pathway can also induce mature miR-21 expression. In this study, we transfected anti-miR-21 oligos as well as the scrambled oligo mocks into human breast cancer cell lines MCF-7 and Hs578T. After 48 hours, with 60%–80% miR-21 knock-down, we observed a significant restoration of MSH2 and SMAD7 mRNA expression (Fig. 6A and 6B). The protein level was increased by ∼35% in MCF-7 and ∼43% in Hs578T for MSH2; and by ∼80% in MCF-7 and ∼133% in Hs578T for SMAD7 by Western blot analysis (Fig. 6C). These findings indicate that overexpression of miR-21 might activate TGF-β signaling by suppressing SMAD7, which functions as an inhibitory SMAD protein in TGF-β signaling. Silencing TGF-β signaling may in turn induce the expression of tumor suppressor genes, such as MSH2.

## Discussion

In this study, we investigated miRNA expression profiling along the linear ductal breast cancer procession model, Normal-ADH-DCIS-IDC, using laser capture microdissected FFPE tissues. The following comparisons were done: ADH vs. Normal, DCIS vs. Normal, and IDC vs. Normal. Analysis revealed that there were more miRNA alterations in the transition between Normal to ADH, suggesting that miRNAs possess a significant role in early tumor initiation; the expression deregulation seems to be maintained throughout DCIS and IDC. These findings agree with previously reported mRNA microarray profiling, which showed that the most prominent transcriptional changes take place at the Normal and ADH stages and such types of alterations could be maintained throughout the later stages [29].

We were unable to readily identify miRNAs that could distinguish between different subgroups at the pre-invasive stages ADH and DCIS, or the invasive stage IDC, as most of the significant alterations of the miRNAs occurred during the normal-ADH transition. These findings might challenge us to rethink our current research viewpoint on the pre-invasive to invasive ductal carcinoma progression. Research on the transition between DCIS to IDC seems to overvalue the focal ductal component, in which selective subpopulations of neoplastic DCIS epithelial cells accumulate with serial genetic alterations and corresponding abilities to disrupt the epithelial layers and then invade from the basement membrane to the surrounding stromal tissues [31] [32]. However, the changes in the microenvironment between DCIS and IDC, in other words, the adjacent non-neoplastic epithelial cells and stromal cells respectively, collaboratively govern a tumor micro-environmental signaling interaction that facilitates the transition from pre-invasive to invasive status. Taken together, the number of ductal carcinoma gene aberrant alteration could not be the only attributor to the DCIS-IDC transition. Without taking the adjacent micro-environment into account, it would be difficult to define the genetic differences between each stage. Nevertheless, we did identify a candidate miRNA, miR-554, which shows a relatively lower expression level exclusively in DCIS stage. This miRNA was identified as significantly altered from both paired and unpaired analysis. This indicates that miR-554 could be a unique miRNA marker for DCIS.

In this study, we also observed one of the currently well studied tumor-suppressor miRNAs, miR-200b, as well as miR-200c from the same family, which showed increased expression throughout all stages. MiR-200b was first reported to directly target E-cadherin repressors ZEB1 and ZEB2 and thus inhibit epithelial-mesenchymal-transition (EMT) in cell line models [33]–[35]. Additional studies show that over-expression of miR-200b/c is able to trigger mesenchymal-epithelial-transition (MET) of metaplastic breast cancer [33]. Ardent investigation and flux of newly published papers suggest that miR-200 families impact cancer invasiveness by collaborating with other molecules, such as Notch [36], Twist1 [37] and PLCγ1 [38]. However, concomitant expression of EMT biomarkers in DCIS compared to IDC revealed that biomarkers including E-cadherin, β-catenin and Snail did not show any statistical significantly positive or negative correlation, except for TGF-β1 and c-Met [39]. On the other hand, miR-200c up-regulation was reported to inhibit pancreatic cancer invasion but increase cell proliferation [26]. This indicates that proliferation is one of the most essential phenotypes of neoplastic cells during the pre-invasive stage. To the best of our knowledge, down-regulation of miR-200 family rein on the tumor precession was not observed in all breast cancer cell lines. For instance, a study in isogenic mouse breast cancer cells indicated that miR-200 members enhance cell colonization to form distant metastases [40]. Additionally, miR-200c was actually observed to be up-regulated to stimulate proliferation in human pancreatic cancer [26]. The function of miR-200 family remains to be elucidated in pre-invasive breast cancer.

Similarly, another well studied miRNA, miR-21 was observed to have displayed an increasing expression trend during breast cancer progression. Target prediction and pathway analysis on the potential downstream targets of miR-21 indicated that miR-21 might promote tumor progression by targeting the TGF-β pathway. MiR-21 can regulate the TGF-β pathway by silencing its inhibitors, such as SMAD7. An up-regulated TGF-β pathway can expedite the generation of mature miR-21 in a feed-forward manner [41], [42]. The nature of TGF-β signaling is controversial, as it can play both a tumor suppressive role by inhibiting cell proliferation and inducing apoptosis in normal epithelial cells, as well as a more aggressive role by promoting tumor growth and invasion. Active TGF-β pathway correlates with poor prognostic and survival rates of breast cancer in the clinic, while suppression of the TGF-β pathway is also reported to be lethal for mice. Therefore it is important to develop strategies to selectively block the cancer-promoting branch but maintain the anti-mitogenic branch of the TGF-β pathway for developing therapeutic drugs. It was recently reported that miR-21 mediates TGF-β bidirectional regulation on MSH2, a central component of DNA mismatch repair (MMR), to contribute chemo-resistance in breast cancer [30]. In the normal cells with intact p53 function and a lower level of miR-21, TGF-β predominantly promotes MSH2 expression, contributing to DNA repair and maintenance of genomic stability. On the other hand, overexpression of miR-21 is often coupled with p53 inactivation in cancerous context, and silences MSH2 by directly binding on its 3′-UTR, resulting in genomic instability and resistance to DNA-damaging chemotherapy agents. MiR-21 might be a mediator for the TGF-β pathway and thus can be a potential target for breast cancer therapy.

In conclusion, deregulation of miRNA expression during tumorigenesis might be an early event as it occurs significantly during normal to ADH transition. Target prediction and pathway analysis revealed that miR-21 has a pivotal role on selective utilization of the TGF-β pathway in breast cancer initiation. Importantly, we have demonstrated the feasibility of miRNA expression profiling analysis using archived FFPE tissues, rich with clinical information, as a means towards miRNA biomarker discovery.

## Materials and Methods

### FFPE Tissue Breast Cancer Samples and Laser Capture Microdissection

A total of 24 female patient breast tissue samples in FFPE from our previous studies [43] were used in this study. Tissue blocks were retrieved from the tissue repository of the Armed Forces Institute of Pathology with IRB approved protocols. Among them, eight were subject to microdissection, resulting in 23 usable tissue components, including normal, hyperplasia, DCIS, and IDC. Different tissue components were separately microdissected from selected cases as described previously [43]. The other 16 FFPE samples with definitive clinical diagnosis of breast lesions were identified, and a total of 4 pieces of 20 µm thick FFPE sections were cut from each case and collected in a 1.5 ml tube.

### RNA Extraction from FFPE Tissue

RecoverAll™ Total Nucleic Acid Isolation Kit for FFPE Tissues (Ambion, Austin, TX) was applied to nucleic acid isolation according to the optimized protocol [44]. Briefly, 1 ml of xylene was added into the 4 pieces of 20 µm thick FFPE sections to remove traces of paraffin. The tissues were digested with protease K at 50°C overnight and treated with DNase I. After washing, total RNA, including a small miRNA fraction, was eluted with distilled water. RNA concentration was measured using the Nanodrop spectrophotometer. The RNA integrity number (RIN) was assessed with an Agilent 2100 Bioanalyzer using the RNA 6000 LabChip kit (Agilent, Palo Alto, CA).

### miRNA Microarray Assay

The Agilent Human MicroRNA Microarray V3 Technology platform was used, which contains 866 mature human miRNAs and 86 viral miRNAs according to the vendor's protocol. Total RNA (100 ng) was dephosphorylated via 37°C incubation with phosphatase for 30 min. The dephosphorylated RNA was mixed with 2.8 µl of 100% DMSO, and then heated at 100°C for 7 min and immediately cooled on an ice water bath. Labeling reactions were carried out and samples incubated at 16°C for 2 hr. Labeled RNA samples were dried in a speed vacuum at 55°C for 3 hours. Samples were then reconstituted and mixed with a hybridization cocktail followed by a 5 min 100°C incubation, and then it was immediately transferred to the ice water bath for 5 min. With samples loaded, hybridization chambers were ensconced in the hybridization oven and incubated at 50°C and a 20 rpm rotation for 20 hr. After hybridization, slides were removed from the chambers and submerged in the provided GE Wash Buffer 1, and washed as follows: GE wash buffer 1 (2 ml 10% Triton X-102 added into 4 L wash buffer) at room temperature for 5 min, and overnight pre-warmed GE Wash Buffer 2 at 37°C for 5 min. Slides were briefly dried and scanned by Agilent's High-Resolution C Scanner.

### Feature Extraction and Pre-processing of miRNA Microarray Data

Probe level data was extracted from the microarray image by using the Agilent Feature Extraction Software (v10.5). QC reports were automatically generated for each array. All the raw data from Feature Extraction were logarithmically transformed to base 2, with quantile algorithm normalization as described [45].

### Statistical Analysis

For the microdissected samples, paired t-test as well as one-way ANOVA were performed to investigate significantly altered miRNAs of one stage against the other three. Deregulated miRNAs were considered as significant if *p*< = 0.05. For the second set of samples, an unpaired t-test as well as one-way ANOVA (Benjamin and Hochberg FDR correction) were performed to identify miRNAs that were changed significantly when comparing one stage against the other three. Each identified miRNA was considered as significant if *p*< = 0.01. A Venn diagram was drawn to show the overlap of miRNAs between the two analyses.

### Hierarchical Clustering Analysis

Unsupervised hierarchical clustering on sample conditions with all detected miRNA entities was generated by Genespring GX 11.5 clustering analysis (Agilent). Euclid distance algorithms were applied for clustering. The unsupervised hierarchical clustering on sample conditions with the most significantly altered miRNA entities was generated in the same manner, while the most significantly altered miRNAs were generated by ANOVA test on all the samples, and filtered by their expression level based on raw data (50^th^ percentile–100^th^ percentile).

### TaqMan miRNA qRT-PCR Analysis

The RT reaction mixture included 10 ng of total RNA as the template, 3 µl 5X RT primer, 1.5 µl 10XRT buffer, 0.15 µl of 100 mM dNTPs, 1 µl of MultiScribe reverse transcriptase, 0.19 µl RNase inhibitor, and 4.16 µl nuclease-free water. The 15 µl reactions were incubated on an ABI 2720 thermal cycler for 30 min at 60°C, 30 min at 42°C, 5 min at 85°C and then held at 4°C. qRT-PCR was performed on an ABI 7300 real-time PCR system. The cocktail of 1.5 µl of 1∶1 diluted RT product, 10 µl Taqman Universal PCR Master Mix with No AmpErase UNG, 7.5 nuclease-free water and 1 µl of 20X MicroRNA Assay were mixed well in an 8-well optical stripe tube, and then incubated according to the following program: 95°C for 10 min, 95°C for 15 sec repeated for 40 cycles, and 60°C for 1 min. All assays were repeated in duplicate with nuclease-free water as the no template control. Relative miRNA expression levels were compared via the 2^−ΔΔCt^ method [46]. To normalize the qPCR results, let-7a was chosen as the reference gene based on its high and consistent expression.

### Target Prediction and Pathway Analysis

Downstream targets of the significantly changed miRNAs were predicted using three different algorithms, specifically TargetScan 6.0, Diana microT 3.0 and miRanda (microrna.org). Each Target was regarded as positive only if it was predicted by at least two of the three algorithms. A list of candidate target genes was subjected to GeneSpring GX or IPA (Ingenuity Pathway Analysis) analysis.

### Anti-miR-21 oligo Transfection

Anti-miR-21 and scrambled mock oligos were purchased from Ambion. On the day of transfection, a total of 240,000 cells were seeded in each well of a 6-well plate. Dilutions of 90 pmol of oligos or mocks and 5 µl of Lipofectamine 2000 (Invitrogen) in 250 µl OptiMEM serum free medium (Invitrogen) were prepared and incubated at room temperature for 20 min. The 500 µl mixture was applied to each well of the 6-well plate. The cells were cultured on antibiotic-free DMEM medium with 10% FBS at a total volume of 3 ml. Cells were harvested after 48 hrs and the miRNAs were isolated using the mirVana miRNA isolation kit (Ambion).

### Western Blot Analysis

The Western blot experiment was performed as described previously [47]. Cell protein lysates were prepared with SDS gel-loading buffer containing β-mercaptoethanol and heated at 95°C for 5 minutes. Proteins were separated by SDS-PAGE using a 12% Mini-PRPTEAN TGX gel (Bio-Rad) and transferred for 2 hours at 100 V. The membrane was blocked prior to the addition of the primary antibody with 5% milk in Tris-buffered saline (TBS) with 0.05% Tween. The membrane was incubated overnight with either MSH2 rabbit polyclonal antibody (Cat# AP11570c, Cell Signaling) at a dilution of 1∶500 in TBS buffer with 0.05% Tween and 5% milk, SMAD7 rabbit polyclonal antibody (Cat# AP6753c, Cell Signaling), at a dilution of 1∶200 in TBS buffer with 0.05% Tween and 5% milk, or GAPDH mouse monoclonal antibody (Cat# MA5-15738, Sigma) at a dilution of 1∶1,000 in TBS buffer with 0.05% Tween. The membrane was washed 3 times with TBS/0.05% Tween and incubated with an anti-rabbit IgG conjugated to horse radish peroxidase (Cat# 7074S, Cell Signaling) for MSH2 and SMAD7, and an anti-mouse IgG (Cat# 7076S, Cell Signaling) for GAPDH at a 1∶1,000 dilution in TBS/0.05% Tween (and 5% milk). Super Signal West Femo Maximum Sensitivity Substrate (Thermo) was used according to the manufacturer's protocol to visualize proteins and quantify band intensity.

## References

[1] Fu SW, Chen L, Man Y-G (2011) miRNA Biomarkers in Breast Cancer Detection and Management. J Cancer 1: 116–122. 10.7150/jca.2.116 PMC3072617 21479130

[2] Lewis F, Maughan NJ, Smith V, Hillan K, Quirke P (2001) Unlocking the archive–gene expression in paraffin-embedded tissue. J Pathol 195: 66–71. 11568892 10.1002/1096-9896(200109)195:1<66::AID-PATH921>3.0.CO;2-F

[3] Masuda N, Ohnishi T, Kawamoto S, Monden M, Okubo K (1999) Analysis of chemical modification of RNA from formalin-fixed samples and optimization of molecular biology applications for such samples. Nucleic Acids Res 27: 4436–4443. 10536153 10.1093/nar/27.22.4436 PMC148727

[4] Xi Y, Nakajima G, Gavin E, Morris CG, Kudo K, et al (2007) Systematic analysis of microRNA expression of RNA extracted from fresh frozen and formalin-fixed paraffin-embedded samples. RNA 13: 1668–1674. 17698639 10.1261/rna.642907 PMC1986820

[5] Li J, Smyth P, Flavin R, Cahill S, Denning K, et al (2007) Comparison of miRNA expression patterns using total RNA extracted from matched samples of formalin-fixed paraffin-embedded (FFPE) cells and snap frozen cells. BMC Biotechnol 7: 36. 17603869 10.1186/1472-6750-7-36 PMC1914054

[6] Hoefig KP, Thorns C, Roehle A, Kaehler C, Wesche KO, et al (2008) Unlocking pathology archives for microRNA-profiling. Anticancer Res 28: 119–123. 18383833

[7] Laios A, O'Toole S, Flavin R, Martin C, Kelly L, et al (2008) Potential role of miR-9 and miR-223 in recurrent ovarian cancer. Mol Cancer 7: 35. 18442408 10.1186/1476-4598-7-35 PMC2383925

[8] Templin MF, Stoll D, Schwenk JM, Potz O, Kramer S, et al (2003) Proteomics 3: 2155–2155. 14595815 10.1002/pmic.200300600

[9] Iorio MV, Ferracin M, Liu CG, Veronese A, Spizzo R, et al (2005) MicroRNA gene expression deregulation in human breast cancer. Cancer Res 65: 7065–7070. 16103053 10.1158/0008-5472.CAN-05-1783

[10] Mattie MD, Benz CC, Bowers J, Sensinger K, Wong L, et al (2006) Optimized high-throughput microRNA expression profiling provides novel biomarker assessment of clinical prostate and breast cancer biopsies. Mol Cancer 5: 24. 16784538 10.1186/1476-4598-5-24 PMC1563474

[11] Wightman B, Ha I, Ruvkun G (1993) Posttranscriptional regulation of the heterochronic gene lin-14 by lin-4 mediates temporal pattern formation in C. elegans. Cell 75: 855–862. 8252622 10.1016/0092-8674(93)90530-4

[12] Valastyan S, Reinhardt F, Benaich N, Calogrias D, Szasz AM, et al (2009) A pleiotropically acting microRNA, miR-31, inhibits breast cancer metastasis. Cell 137: 1032–1046. 19524507 10.1016/j.cell.2009.03.047 PMC2766609

[13] Tavazoie SF, Alarcon C, Oskarsson T, Padua D, Wang Q, et al (2008) Endogenous human microRNAs that suppress breast cancer metastasis. Nature 451: 147–152. 18185580 10.1038/nature06487 PMC2782491

[14] Tsuchiya Y, Nakajima M, Takagi S, Taniya T, Yokoi T (2006) MicroRNA regulates the expression of human cytochrome P450 1B1. Cancer Res 66: 9090–9098. 16982751 10.1158/0008-5472.CAN-06-1403

[15] Zhang J, Du YY, Lin YF, Chen YT, Yang L, et al (2008) The cell growth suppressor, mir-126, targets IRS-1. Biochem Biophys Res Commun 377: 136–140. 18834857 10.1016/j.bbrc.2008.09.089

[16] Esquela-Kerscher A, Slack FJ (2006) Oncomirs - microRNAs with a role in cancer. Nat Rev Cancer 6: 259–269. 16557279 10.1038/nrc1840

[17] Manikandan J, Aarthi JJ, Kumar SD, Pushparaj PN (2008) Oncomirs: the potential role of non-coding microRNAs in understanding cancer. Bioinformation 2: 330–334. 18685719 10.6026/97320630002330 PMC2478731

[18] Frankel LB, Christoffersen NR, Jacobsen A, Lindow M, Krogh A, et al (2008) Programmed cell death 4 (PDCD4) is an important functional target of the microRNA miR-21 in breast cancer cells. J Biol Chem 283: 1026–1033. 17991735 10.1074/jbc.M707224200

[19] Kong W, Yang H, He L, Zhao JJ, Coppola D, et al (2008) MicroRNA-155 is regulated by the transforming growth factor beta/Smad pathway and contributes to epithelial cell plasticity by targeting RhoA. Mol Cell Biol 28: 6773–6784. 18794355 10.1128/MCB.00941-08 PMC2573297

[20] Ma L, Teruya-Feldstein J, Weinberg RA (2007) Tumour invasion and metastasis initiated by microRNA-10b in breast cancer. Nature 449: 682–688. 17898713 10.1038/nature06174

[21] Huang Q, Gumireddy K, Schrier M, le Sage C, Nagel R, et al (2008) The microRNAs miR-373 and miR-520c promote tumour invasion and metastasis. Nat Cell Biol 10: 202–210. 18193036 10.1038/ncb1681

[22] Mertens-Talcott SU, Chintharlapalli S, Li X, Safe S (2007) The oncogenic microRNA-27a targets genes that regulate specificity protein transcription factors and the G2-M checkpoint in MDA-MB-231 breast cancer cells. Cancer Res 67: 11001–11011. 18006846 10.1158/0008-5472.CAN-07-2416

[23] Miller TE, Ghoshal K, Ramaswamy B, Roy S, Datta J, et al (2008) MicroRNA-221/222 confers tamoxifen resistance in breast cancer by targeting p27Kip1. J Biol Chem 283: 29897–29903. 18708351 10.1074/jbc.M804612200 PMC2573063

[24] Hsiao YH, Tsai HD, Chou MC, Man YG (2011) The myoepithelial cell layer may serve as a potential trigger factor for different outcomes of stage-matched invasive lobular and ductal breast cancers. Int J Biol Sci 7: 147–153. 21326853 10.7150/ijbs.7.147 PMC3039295

[25] Ladeiro Y, Couchy G, Balabaud C, Bioulac-Sage P, Pelletier L, et al (2008) MicroRNA profiling in hepatocellular tumors is associated with clinical features and oncogene/tumor suppressor gene mutations. Hepatology 47: 1955–1963. 18433021 10.1002/hep.22256

[26] Yu J, Ohuchida K, Mizumoto K, Sato N, Kayashima T, et al (2010) MicroRNA, hsa-miR-200c, is an independent prognostic factor in pancreatic cancer and its upregulation inhibits pancreatic cancer invasion but increases cell proliferation. Mol Cancer 9: 169. 20579395 10.1186/1476-4598-9-169 PMC2909980

[27] Heneghan HM, Miller N, Kelly R, Newell J, Kerin MJ (2010) Systemic miRNA-195 differentiates breast cancer from other malignancies and is a potential biomarker for detecting noninvasive and early stage disease. Oncologist 15: 673–682. 20576643 10.1634/theoncologist.2010-0103 PMC3228012

[28] Hannafon BN, Sebastiani P, de Las Morenas A, Lu J, Rosenberg CL (2011) Expression of microRNAs and their gene targets are dysregulated in pre-invasive breast cancer. Breast Cancer Res 13: R24. 21375733 10.1186/bcr2839 PMC3219184

[29] Ma XJ, Salunga R, Tuggle JT, Gaudet J, Enright E, et al (2003) Gene expression profiles of human breast cancer progression. Proc Natl Acad Sci U S A 100: 5974–5979. 12714683 10.1073/pnas.0931261100 PMC156311

[30] Yu Y, Wang Y, Ren X, Tsuyada A, Li A, et al (2010) Context-dependent bidirectional regulation of the MutS homolog 2 by transforming growth factor beta contributes to chemoresistance in breast cancer cells. Mol Cancer Res 8: 1633–1642. 21047769 10.1158/1541-7786.MCR-10-0362 PMC3059495

[31] Man YG, Sang QX (2004) The significance of focal myoepithelial cell layer disruptions in human breast tumor invasion: a paradigm shift from the “protease-centered” hypothesis. Exp Cell Res 301: 103–118. 15530847 10.1016/j.yexcr.2004.08.037

[32] Sasaki M, Ikeda H, Haga H, Manabe T, Nakanuma Y (2005) Frequent cellular senescence in small bile ducts in primary biliary cirrhosis: a possible role in bile duct loss. J Pathol 205: 451–459. 15685690 10.1002/path.1729

[33] Gregory PA, Bert AG, Paterson EL, Barry SC, Tsykin A, et al (2008) The miR-200 family and miR-205 regulate epithelial to mesenchymal transition by targeting ZEB1 and SIP1. Nat Cell Biol 10: 593–601. 18376396 10.1038/ncb1722

[34] Korpal M, Lee ES, Hu G, Kang Y (2008) The miR-200 family inhibits epithelial-mesenchymal transition and cancer cell migration by direct targeting of E-cadherin transcriptional repressors ZEB1 and ZEB2. J Biol Chem 283: 14910–14914. 18411277 10.1074/jbc.C800074200 PMC3258899

[35] Park SM, Gaur AB, Lengyel E, Peter ME (2008) The miR-200 family determines the epithelial phenotype of cancer cells by targeting the E-cadherin repressors ZEB1 and ZEB2. Genes Dev 22: 894–907. 18381893 10.1101/gad.1640608 PMC2279201

[36] Brabletz S, Bajdak K, Meidhof S, Burk U, Niedermann G, et al (2011) The ZEB1/miR-200 feedback loop controls Notch signalling in cancer cells. EMBO J 30: 770–782. 21224848 10.1038/emboj.2010.349 PMC3041948

[37] Wiklund ED, Bramsen JB, Hulf T, Dyrskjot L, Ramanathan R, et al (2010) Coordinated epigenetic repression of the miR-200 family and miR-205 in invasive bladder cancer. Int J Cancer 128: 1327–1334. 10.1002/ijc.25461 20473948

[38] Uhlmann S, Zhang JD, Schwager A, Mannsperger H, Riazalhosseini Y, et al (2010) miR-200bc/429 cluster targets PLCgamma1 and differentially regulates proliferation and EGF-driven invasion than miR-200a/141 in breast cancer. Oncogene 29: 4297–4306. 20514023 10.1038/onc.2010.201

[39] Logullo AF, Nonogaki S, Pasini FS, Osorio CA, Soares FA, et al (2010) Concomitant expression of epithelial-mesenchymal transition biomarkers in breast ductal carcinoma: association with progression. Oncol Rep 23: 313–320. 20043090

[40] Dykxhoorn DM, Wu Y, Xie H, Yu F, Lal A, et al (2009) miR-200 enhances mouse breast cancer cell colonization to form distant metastases. PLoS One 4: e7181. 19787069 10.1371/journal.pone.0007181 PMC2749331

[41] Davis-Dusenbery BN, Hata A (2011) Smad-mediated miRNA processing: a critical role for a conserved RNA sequence. RNA Biol 8: 71–76. 21289485 10.4161/rna.8.1.14299 PMC3230544

[42] Davis BN, Hilyard AC, Nguyen PH, Lagna G, Hata A (2010) Smad proteins bind a conserved RNA sequence to promote microRNA maturation by Drosha. Mol Cell 39: 373–384. 20705240 10.1016/j.molcel.2010.07.011 PMC2921543

[43] Arroyo JD, Chevillet JR, Kroh EM, Ruf IK, Pritchard CC, et al (2011) Argonaute2 complexes carry a population of circulating microRNAs independent of vesicles in human plasma. Proc Natl Acad Sci U S A 108: 5003–5008. 21383194 10.1073/pnas.1019055108 PMC3064324

[44] Abramovitz M, Ordanic-Kodani M, Wang Y, Li Z, Catzavelos C, et al (2008) Optimization of RNA extraction from FFPE tissues for expression profiling in the DASL assay. Biotechniques 44: 417–423. 18361796 10.2144/000112703 PMC2672087

[45] Bolstad BM, Irizarry RA, Astrand M, Speed TP (2003) A comparison of normalization methods for high density oligonucleotide array data based on variance and bias. Bioinformatics 19: 185–193. 12538238 10.1093/bioinformatics/19.2.185

[46] Livak KJ, Schmittgen TD (2001) Analysis of relative gene expression data using real-time quantitative PCR and the 2(-Delta Delta C(T)) Method. Methods 25: 402–408. 11846609 10.1006/meth.2001.1262

[47] Kluk BJ, Fu Y, Formolo TA, Zhang L, Hindle AK, et al (2010) BP1, an isoform of DLX4 homeoprotein, negatively regulates BRCA1 in sporadic breast cancer. Int J Biol Sci 6: 513–524. 20877436 10.7150/ijbs.6.513 PMC2945279

